# Supplementary material for: A case study for a psychographic-behavioral segmentation approach for targeted demand generation in voluntary medical male circumcision
Source: eLife. 2017 Sep 13;6:e25923. doi: 10.7554/eLife.25923 (PMC5628013; doi:10.7554/eLife.25923)
Supplement: Figure 4—source data 1. [file elife-25923-fig4-data1.docx]

| **Country** | **Question** | | **Scale** |
| --- | --- | --- | --- |
| **Zimbabwe** | 1 | I am committed to getting circumcised | 7-point scale: 7 = 'Strongly agree'; 4 = 'Neither agree nor disagree'; 1 = 'Strongly disagree' |
|  | 2 | I understand the procedure and healing process for circumcision | 7-point scale: 7 = 'Strongly agree'; 4 = 'Neither agree nor disagree'; 1 = 'Strongly disagree' |
|  | 3 | I am embarrassed to go get circumcised | 7-point scale: 7 = 'Strongly agree'; 4 = 'Neither agree nor disagree'; 1 = 'Strongly disagree' |
|  | 4 | It is our duty towards society to prevent HIV | 7-point scale: 7 = 'Strongly agree'; 4 = 'Neither agree nor disagree'; 1 = 'Strongly disagree' |
|  | 5 | Even if my friends or people in my community may come to know I am circumcised, I would get circumcised if I wanted | 7-point scale: 7 = 'Strongly agree'; 4 = 'Neither agree nor disagree'; 1 = 'Strongly disagree' |
|  | 6 | Going for circumcision is too stressful | 7-point scale: 7 = 'Strongly agree'; 4 = 'Neither agree nor disagree'; 1 = 'Strongly disagree' |
|  | 7 | How likely would you be to encourage your grandfather to go for male circumcision? | 7-point scale: 7 = 'Would definitely encourage'; 4 = 'Would neither encourage nor discourage'; 1 = 'Would definitely NOT encourage' |
|  | 8 | How likely would you be to encourage your son (if you have or had one) to go for male circumcision? | 7-point scale: 7 = 'Would definitely encourage'; 4 = 'Would neither encourage nor discourage'; 1 = 'Would definitely NOT encourage' |
|  | 9 | What do or what would other female family members think about your decision if you were to get circumcised? | 7-point scale: 7 = 'They think I definitely should get circumcised'; 4 = 'They don't have any particular opinion'; 1 = 'They think I definitely should NOT get circumcised' |
| **Zambia** | 1 | I can envision myself getting circumcised | 7-point scale: 7 = 'Strongly agree'; 4 = 'Neither agree nor disagree'; 1 = 'Strongly disagree' |
|  | 2 | How likely would you be to encourage a brother (if you have or had one) to go for male circumcision? | 7-point scale: 7 = 'Would definitely encourage'; 4 = 'Would neither encourage nor discourage'; 1 = 'Would definitely NOT encourage' |
|  | 3 | How likely would you be to encourage your grandfather to go for male circumcision? | 7-point scale: 7 = 'Would definitely encourage'; 4 = 'Would neither encourage nor discourage'; 1 = 'Would definitely NOT encourage' |
|  | 4 | It would be easier for me to get circumcised if no surgery or stitches are required | 7-point scale: 7 = 'Strongly agree'; 4 = 'Neither agree nor disagree'; 1 = 'Strongly disagree' |
|  | 5 | Getting circumcised is my own decision | 7-point scale: 7 = 'Strongly agree'; 4 = 'Neither agree nor disagree'; 1 = 'Strongly disagree' |
|  | 6 | Level of belief in the benefits of male circumcision | 5-point scale: 5 = 'There definitely are benefits of male circumcision'; 3 = 'I am not sure whether there are benefits of male circumcision'; 1 = 'There definitely are NO benefits of male circumcision' |
|  | 7 | I understand the procedure and healing process for circumcision | 7-point scale: 7 = 'Strongly agree'; 4 = 'Neither agree nor disagree'; 1 = 'Strongly disagree' |
|  | 8 | Going to get circumcised would be an easy thing for me to do | 7-point scale: 7 = 'Strongly agree'; 4 = 'Neither agree nor disagree'; 1 = 'Strongly disagree' |

**Figure 4 – source data 1. Segment typing tool questions**
